# Fracture Lower Jaw in an Infant

**Published:** 1878-03

**Authors:** Geo. A. Mursick

**Affiliations:** Nyack, N. Y.


Dear Doctor:—The following case is sufficiently unusual to demand a permanent record, and I have for this purpose sent it to you. Very Truly Yours,

FRANK H. HAMILTON.

Nyack, N. Y. Feb. 14th 1878.

*Fracture of the Lower Jaw in an infant.—Fragments maintained in position by wire suture.*

Dr. F. H. Hamilton

Dear Sir:—There does not appear on record many reports of cases of Fracture of the Inferior Maxilla, treated by wire sutures. I therefore infer that this method of treatment has not been much practiced. Knowing the great interest you take in the matter of fractures generally, I have thought the history of the following case might be of interest to you.

August 3d, 1876.—Williams, a boy aged three years, fell from the third story window of the house in which he lived, to the street, a distance of about thirty feet. I saw him an hour afterwards. He was then insensible, suffering from concussion etc. Upon examination I found a simple fracture of the inferior maxilla through the middle of the horizontal rhamus of the right side. Other than this there was not a scratch or bruise upon him. No dressing was applied until the next day, when he had in a great measure recovered from the shock. There was not much displacement of the bones. To prevent his interfering with the dressings I applied adhesive strips to the jaw in the shape of a Boston bandage. This fixed the jaw firm and immovable. Everything did well until—

August 29th, when he fell down a flight of stairs and refractured his jaw. The end of the *anterior* fragment of the bone was driven through the integument of the cheek and overlapped the front of the posterior fragment of the bone about one-half an inch. After some difficulty the ends of the bone were brought in apposition but could not be so retained by any external appliance.

After anesthesia by ether, I dissected the inside of the cheek from the ends of the bone, bored a hole through each end with a bradawl, passed a stout silver wire suture through, and twisted it tight. This brought the ends of the bone in perfect apposition, and held them securely. The ends of the wire were brought out through the external wound; this served to facilitate free drainage, as well as the final removal of the suture. After three weeks the fracture was finaly united, and two weeks later the suture came away, after which the external wound soon healed. The teeth are level and the articulation perfect. With the exception of some flattening of that side of the face there is no deformity.

Very Truly Yours, &c.,

GEO. A. MURSICK, M. D.

